# Demographic features, clinical characteristics, and comorbid relation in hidradenitis suppurativa: a population-based study

**DOI:** 10.3389/fmed.2024.1499509

**Published:** 2025-01-09

**Authors:** Gökhan Kaya, Fatma Pelin Özgen, Osman Kelahmetoğlu, Özlem Su Küçük, Nahide Onsun

**Affiliations:** ^1^Ministry of Health Nizip State Hospital, Department of Dermatology, Gaziantep, Türkiye; ^2^Department of Dermatology, Üsküdar University, İstanbul, Türkiye; ^3^Istanbul Okan University, Department of Plastic, Reconstructive, and Aesthetic Surgery, Istanbul, Türkiye; ^4^Department of Dermatology, Faculty of Medicine, Bezmialem Vakif University, Istanbul, Türkiye; ^5^Department of Dermatology, Biruni University, İstanbul, Türkiye

**Keywords:** hidradenitis suppurativa, epidemiology, clinical characteristics, severity, Hurley, comorbidities

## Abstract

**Background/objective:**

Hidradenitis suppurativa (HS) is a chronic inflammatory disease affecting apocrine gland areas, characterized by painful nodules and abscesses that may result in sinus tracts and scarring. The global prevalence of HS is increasing due to heightened awareness, improved diagnostic methods, rising obesity rates, and higher smoking prevalence. This study aimed to describe the epidemiological, clinical, and comorbid characteristics of HS patients.

**Methodology:**

This retrospective descriptive cross-sectional study included 193 outpatients aged between 15 and 73 years who visited a tertiary HS clinic between 2017 and 2022. Demographic, clinical, and comorbid characteristics were recorded and analyzed using chi-square and ordinal regression methods.

**Results:**

The mean age was 34.5 ± 12.1 years, with a mean disease duration of 5.9 ± 6.7 years. According to the Hurley classification, 61.1, 24.4, and 14.5% were stages I, II, and III, respectively. Comorbidities were present in 48.2% of patients, with psychiatric disorders (19.2%), diabetes mellitus (14%), and hypertension (9.3%) being most common. Disease duration, smoking, male gender, and atypical localization were associated with increased disease severity.

**Conclusion:**

Geographic and cultural factors influence the prevalence, severity, and management of HS, necessitating tailored treatment. Effective management requires multidisciplinary screening for early detection and prevention of comorbidities, including psychiatric disorders, cardiovascular conditions, and metabolic syndrome.

## 1 Introduction

Hidradenitis suppurativa (HS), also known as acne inversa, is a recurrent, painful chronic inflammatory skin disease that affects approximately 1% of the population. Although HS typically occurs after puberty, it is often observed in the second to third decades of life, with a slight female predominance (1). Many theories have been proposed regarding its pathogenesis; however, inflammation, genetic susceptibility, and bacterial growth are the most frequently mentioned mechanisms (2). HS has significant morbidity, and numerous tests to measure quality of life indicate that patients with HS experience significant physical, social, and emotional problems compared with many other dermatological diseases (3). Early diagnosis may be important for disease management, as delays in diagnosis or mismanagement of treatment may lead to the development of multiple comorbidities (4). Many comorbidities associated with HS, such as metabolic syndrome, poor quality of life, sexual dysfunction, difficulty working, inflammatory bowel disease, axial spondyloarthritis, depression, and anxiety, have been described, and a multidisciplinary approach is important for holistic treatment of HS (5).

This study investigates the epidemiology, clinical characteristics, and comorbidities of HS patients at our center, aiming to improve our understanding and treatment of a condition that significantly compromises quality of life.

## 2 Methods

### 2.1 Study design and study population

This observational, retrospective, cross-sectional study included consecutive 193 patients diagnosed with HS at the Dermatology Outpatient Clinic of a tertiary university hospital in Türkiye between January 2017 and December 2022. During this period, patients with HS were seen in a dedicated clinic held on specific days of the week, ensuring focused and consistent care for this patient population. Diagnoses followed the International Hidradenitis Suppurativa Diagnosis and Treatment Guidelines, requiring painful nodules, abscesses, and sinus tracts to recur at least twice within 6 months, and only patients meeting these criteria were included. Initially, 230 patients were identified for the study; however, 37 were excluded due to incomplete medical records (15 patients), loss to follow-up (12 patients), or failure to meet the established diagnostic criteria (10 patients).

### 2.2 Ethical approval statement

This study was reviewed and approved by the Non-Interventional Research Ethics Committee of Bezmialem Vakıf University (Approval Number: E-54022451-050.05.04-80602, 2022/296). As the study was retrospective, the ethics committee waived the requirement for informed consent. This study was conducted in accordance with the principles of the Declaration of Helsinki.

### 2.3 Clinical and laboratory data collection

Clinical and laboratory information was documented using the hospital’s digital data recording system. Collected data included demographics (age and gender), clinical characteristics (age at diagnosis, duration of disease, delay in diagnosis), family history of HS, and the presence of the follicular occlusion tetrad (FOT) including dissecting cellulitis of the scalp (DCS), acne conglobata (AC), and pilonidal disease (PD). Biochemical tests, complete blood counts, and infection parameters were obtained to guide treatment decisions and to identify comorbid conditions. Patients exhibiting abnormal values or suspected comorbidities were referred to relevant specialties with documented conditions such as cardiovascular diseases, diabetes, and dyslipidemia. Psychiatric comorbidities such as anxiety and depression were identified through previous diagnoses in patient records, utilizing scales like the Beck Anxiety Inventory to determine their presence. Clinical images were captured using smartphones and uploaded to the digital cloud system at each visit, facilitating the monitoring of clinical changes due to treatment. Disease severity was assessed using the Hurley staging system to classify patients into Hurley stages I, II, and III for subsequent treatment planning.

### 2.4 Patient questionnaire and self-reported data

In the questionnaire, participants self-reported their weight, height, living habits (including smoking status and alcohol use), and opinions about their current health status. Body mass index (BMI) was calculated as the ratio of weight to height squared (kg/m^2^). Study participants were classified into four groups according to BMI and World Health Organization (WHO) criteria: underweight (<18.5 kg/m^2^), normal weight (18.5–25 kg/m^2^), overweight (25–30 kg/m^2^), and obese (≥30 kg/m^2^). Smoking status was assessed by calculating the number of cigarette packs per year. Alcohol use was categorized as never, rarely, weekly or daily. Participants also reported a history of other diseases, both somatic and psychiatric. Socioeconomic status was determined on the basis of educational level and employment status. Education was categorized as primary school, middle school, high school, undergraduate, or postgraduate. Employment status was recorded as full-time employment or student due to illness, retirement, or unemployment. The symptoms recorded included pain, discomfort, itching, swelling, and fever. Participants reported both the presence and frequency of these symptoms as well as the frequency of flare-ups, categorized as none, 1–2 times per year, more than 3 times per year, 1–2 times per month, and 4–5 times per month.

### 2.5 Statistical analysis

Quantitative data were described using means, standard deviations, and ranges after preliminary data cleaning which involved checking for outliers and ensuring data integrity. Qualitative data were expressed as frequencies and percentages. The Shapiro–Wilk test confirmed that the quantitative data did not follow a normal distribution, prompting the use of non-parametric tests for analysis. Specific reasons for choosing non-parametric tests include their suitability for handling skewed data distributions and ordinal data.

Chi-square tests were used to evaluate associations between categorical variables, with the Bonferroni correction applied to adjust for multiple comparisons and minimize Type I errors. For continuous variables, the Student’s t-test provided a means to compare means between two groups, selected due to the robustness of this test in handling non-normal data when sample sizes are sufficiently large.

Further, univariate and multivariate ordinal regression analyses explored factors affecting the severity of Hurley’s disease classification. Variable selection in the multivariate models employed a backward stepwise approach based on likelihood ratios, with variables retained at a significance level of *p* < 0.10 to ensure a comprehensive model. Missing data points were handled using pairwise deletion, which was deemed appropriate given the low incidence of missing values.

All analyses were performed using IBM SPSS Statistics software (version 26), ensuring a rigorous statistical framework. The significance threshold was set at 0.05, balancing the risk of Type I and Type II errors.

## 3 Results

This study included a cohort of 193 patients diagnosed with HS, including 121 males (62.7%) and 72 females (37.3%), with an age range of 15–73 years. The average age was 34.5 years, with a mean age at diagnosis of 28.8 years. The mean disease duration was 6 years, and the average diagnostic delay was 4.3 years. No significant gender differences were observed in terms of age, age at diagnosis, and disease duration. The mean BMI was 27.84 kg/m^2^, with 32.8% of patients categorized as normal weight, 32.8% as overweight, and 34.4% as obese. Significant gender differences were observed: a higher proportion of females were in the normal weight category (45.1 vs. 25.6%), while males were more likely to be overweight (38.0 vs. 23.9%; *p* = 0.064). Smoking prevalence was higher in males (*p* < 0.001). Males were more likely to be full-time employees or students (*p* < 0.001), while the rate of being unable to work due to illness did not differ significantly between males and females. Common symptoms included pain (74.6%), swelling (76.7%), and discomfort (71.5%), with no significant gender differences. The intermittent periods of worsening varied, with 25.4% experiencing more than three such periods per year, and there were no significant differences between males and females (*p* = 0.235). A family history of HS was noted in 21.8% of patients, with no significant gender difference (*p* = 0.279). Hospitalization occurred in 18.7% of patients, with similar rates between genders (*p* ≈ 1.0; Table 1).

**Table 1:** Demographic and clinical characteristics of the study population.

Parameters	All patients (*n* = 193)	Female (*n* = 72)	Male (*n* = 121)	*p* value
Age (years), mean ± SD (range)	34,51 ± 12,11 (15–73)	32,78 ± 12,24 (16–68)	35,55 ± 11,97 (15–73)	0,125
Age at Diagnosis (years), mean ± SD (range)	28,75 ± 12,11 (11–71)	27,69 ± 12,58 (11–66)	29,38 ± 11,84 (13–71)	0,351
Duration of Disease (years), mean ± SD (range)	5,94 ± 6,65 (0–55)	5,05 ± 4,98 (1–22)	6,46 ± 7,44 (0–55)	0,154
Delay in Diagnosis (years), mean ± SD (range)	4,31 ± 5,64 (0–34)	3,76 ± 4,91 (0–19)	4,64 ± 6,03 (0–34)	0,300
BMI (kg/m^2^), mean ± SD (range)	27,84 ± 5,25 (16,8-47,3)	26,87 ± 5,96 (16,8-42,5)	28,42 ± 4,72 (19,5-47,3)	0,064
BMI Categories (%)	Normal: 63 (32,8%)	Normal: 32^a^ (45,1%)	Normal: 31^b^ (25,6%)	**0,016**
	Overweight: 63 (32,8%)	Overweight: 17^a^ (23,9%)	Overweight: 46^b^ (38%)	
	Obese: 66 (34,4%)	Obese: 22^a^ (31%)	Obese: 44^a^ (36.4%)	
Area Retained ± SD, (min–max)	2,55 ± 1,34 (1–7)	2,47 ± 1,32 (1–6)	2,6 ± 1,35 (1–7)	0,539
HS Family History *n*,%	42 (21,8%)	19 (26,4%)	23 (19%)	0,279
Cigarettes (pack/year) ± SD, (min–max)	11,39 ± 11,99 (0–60)	7,17 ± 9,9 (0–40)	13,91 ± 12,45 (0–60)	**<0,001**
Alcohol Use	Never: 117 (60.6%),	Never: 53^a^ (73.6%),	Never: 64^b^ (52.9%),	**0,023**
	Rarely: 51 (26.4%),	Rarely: 12^a^ (16.7%),	Rarely: 39^b^ (32.2%),	
	Weekly: 21 (10.9%),	Weekly: 5^a^ (6.9%),	Weekly: 16^a^ (13.2%),	
	Daily: 4 (2.1%)	Daily: 2^a^ (2.8%)	Daily: 2^a^ (1.7%)	
Employment Status (%)	Full-time/Student: 145 (75.1%)	Full-time/Student: 42^a^ (58,3%)	Full-time/Student: 103^b^ (85,1%)	**<0,001**
	Due to Illness: 11 (5.7%)	Due to Illness: 4^a^ (5,6%)	Due to Illness: 7^a^ (5,8%)	
	Retired: 7 (3.6%)	Retired: 0^a^ (0%)	Retired: 7^b^ (5,8%)	
	Unemployed: 30 (15.5%)	Unemployed: 26^a^ (36,1%)	Unemployed: 4^b^ (3,3%)	
Education Level *n* (%)	Primary School: 35 (18.1%)	Primary School: 15 (20.8%)	Primary School: 20 (16.5%)	0,341
	Middle School: 13 (6.7%)	Middle School: 2 (2.8%)	Middle School: 11 (9.1%)	
	High School: 55 (28.5%)	High School: 18 (25%)	High School: 37 (30.6%)	
	Undergraduate: 80 (41.5%)	Undergraduate: 32 (44.4%)	Undergraduate: 48 (39.7%)	
	Postgraduate: 10 (5.2%)	Postgraduate: 5 (6.9%)	Postgraduate: 5 (4.1%)	
Symptoms	Pain: 144 (74.6%),	Pain: 49 (68,1%),	Pain: 95 (78,5%),	**Pain**: 0,125,
	Discomfort: 138 (71.5%),	Discomfort: 50 (69,4%)	Discomfort: 88 (72,7%),	**Discomfort**: 0,742
	Itching: 99 (51.3%),	Itching: 39 (54,2%),	Itching: 60 (49,6%),	**Itching**: 0,555
	Swelling: 48 (76.7%),	Swelling: 50 (69,4%),	Swelling: 98 (81%),	**Swelling**: 0,079
	Fever: 71 (36.8%)	Fever: 31 (43,1%)	Fever: 40 (33,1%)	**Fever**: 0,169
Intermittent Periods of Worsening (Flare)	None: 31 (16.1%)	None: 14 (19.4%)	None: 17 (14%)	0,235
	1–2 times/year: 40 (20.7%)	1–2 times/year: 12 (16.7%)	1–2 times/year: 28 (23.1%)	
	>3 times/year: 49 (25.4%)	>3 times/year: 15 (20.8%)	>3 times/year: 34 (28.1%)	
	1–2 times/month: 50 (25.9%)	1–2 times/month: 24 (33.3%)	1–2 times/month: 26 (21.5%)	
	4–5 times/month: 23 (11.9%)	4–5 times/month: 7 (9.7%)	4–5 times/month: 16 (13.2%)	
Acne Vulgaris	Never: 82 (42.5%)	Never: 34^a^ (47.2%),	Never: 48^a^ (39.7%),	**0,025**
	Past: 65 (33.7%),	Past: 16^a^ (22.2%),	Past: 49^b^ (40.5%),	
	Current: 46 (23.8%)	Current: 22^a^ (30.6%)	Current: 24^a^ (19.8%)	
Follicular Occlusion Tetrad (FOT) Prevalence (%)	DCS: 13 (6.7%)	DCS: 1 (1.4%)	DCS: 12 (9.9%)	**DCS: 0,034**
	AC: 21 (10.9%)	AC: 2 (2.8%)	AC: 19 (15.7%)	**AC: 0,007**
	PD: 69 (35.8%)	PD: 15 (20.8%)	PD: 54 (44.6%)	**PD: 0,001**
Hurley Stage Distribution (%)	I: 118 (61,1%)	I: 54^a^ (75%)	I: 64^b^ (52,9%)	**0,007**
	II: 47 (24,4%)	II: 13^a^ (18,1%)	II: 34^a^ (28,1%)	
	III: 28 (14,5%)	III: 5^a^ (6,9%)	III: 23^b^ (19%)	
Hospitalization Rate (%)	36 (18,7%)	13^a^ (18,1%)	23 (19%)	≈1,0

The demographic and clinical characteristics of the patients, including age, gender distribution, age at diagnosis, duration of disease, delay in diagnosis, and BMI are presented. Additionally, familial history of HS and lifestyle factors, such as smoking and alcohol use, were also included (the symbols ^a^ and ^b^ are used to separate statistically significant variables from multiple variables). The bold values are indicate statistically significant results, with a *p*-value less than 0.05.

### 3.1 Associated diseases

Comorbidities revealed psychiatric disorders in 19.2% of patients, with no significant gender difference (p ≈ 1.0). Cardiovascular disease and diabetes mellitus (DM) were found in 4.7 and 14% of patients, respectively, showing a similar prevalence across genders. Dyslipidemia (6.7%) and hypertension (9.3%) were also common, without significant gender disparities. Inflammatory bowel disease (IBD) was observed in six patients (3.1%), including five with Crohn’s disease (CD) and one with ulcerative colitis (UC). Eight patients (4.1%) had spondyloarthritis (SpA). Due to the small sample size, the results for chronic kidney disease and genetic disorders should be interpreted cautiously. These findings highlight the range of comorbidities associated with HS and emphasize the need for comprehensive patient care (Table 2).

**Table 2:** Comorbidities in patients with hidradenitis suppurativa

Comorbidities, n (%)	All Patients *n* = 93 (48,2%)	Female *n* = 35 (48,6%)	Male *n* = 58 (47,9%)	*p* value
Psychiatric Disorders	37 (19,2%)	14 (19,4%)	23 (19%)	≈1,0
Cardiovascular Disease	9 (4,7%)	2 (2,8%)	7 (5,8%)	0,488
Systemic Hypertension	18 (9,3%)	5 (6,9%)	13 (10,7%)	0,451
Diabetes Mellitus with Insulin Resistance	27 (14%)	10 (13,9%)	17 (14%)	≈1,0
Dyslipidemia	13 (6,7%)	3 (4,2%)	10 (8,3%)	0,378
Spondyloarthropathies	8 (4,1%)	3 (4,2%)	5 (%)	≈1,0
Chronic Kidney Disease	4 (2,1%)	0 (0%)	4 (3,3%)	0,299
Neoplastic Disorders	4 (2,1%)	2 (2,8%)	2 (1,7%)	0,630
Genetic Disease	1 (0,5%)	1 (1,4%)	0 (0%)	0,373
Polycystic Ovary Syndrome (PCOS)	1 (0,5%)	1 (1,4%)	0 (0%)	0,373
Asthma	5 (2,6%)	3 (4,2%)	2 (1,7%)	0,364
Inflammatory Bowel Diseases	6 (3,1%)	3 (4,2%)	3 (2,5%)	0,673
Chron’s Disease(CD)	CD: 5	CD: 3	CD: 2	
Ulcerative Colitis(UC)	UC: 1	UC: 0	UC: 1	
Autoimmune Thyroiditis	2 (1%)	2 (2,8%)	0 (0%)	0,138
Non-alcoholic Fatty Liver Disease (NAFLD)	2 (1%)	1 (1,4%)	1 (0,8%)	≈1,0
Psoriasis	5 (2,6%)	2 (2,8%)	3 (2,5%)	≈1,0
Familial Mediterranean Fever (FMF)	4 (2,1%)	2 (2,8%)	2 (1,7%)	0,630
Cerebrovascular accident (CVA)	1 (0,5%)	0 (0%)	1 (0,8%)	≈1,0

The prevalence of various comorbid conditions among the patients, including psychiatric disorders, cardiovascular disease, hypertension, diabetes mellitus, and other significant health issues, is detailed.

### 3.2 Acne vulgaris and follicular occlusion tetrad in HS patients

In our cohort, 42.5% of patients did not have acne vulgaris, 33.7% had a history of acne, and 23.8% had current acne. The prevalence rates of FOT were as follows: DCS (6.7%), AC (10.9%), and PD (35.8%). Notably, only five patients met all criteria for the tetrad, with significantly higher rates of each component observed in men than in women (*p* = 0.025).

### 3.3 Lesion involvement areas

Analysis of the lesion distribution in patients with HS revealed significant gender differences. The average lesion involvement area across the entire body was 2.55 units, with no significant difference between females (2.47 units) and males (2.6 units; *p* = 0.539). Axillary involvement was observed in 80.8% of the patients, with 75% of females and 84.3% of males affected. Groin involvement was higher in females (70.8%) compared to males (59.5%). Chest involvement was more prevalent in females (31.9%) than in males (13.2%, *p* = 0.003), whereas abdominal involvement was more common in males (20.7%) than in females (6.9%, *p* = 0.013). Due to the small sample sizes for atypical localization areas such as the leg and posterior neck regions, these results should be interpreted with caution (Table 3).

**Table 3:** Lesion distribution by gender.

Lesion area	All Regions *n* (%)	% to 95 CI	Female, *n* (%)	Male, *n*(%)	*p* value
Axilla	156 (80,8%)	75,2%; 86,4%	54 (75%)	102 (84,3%)	0,132
Groin	123 (63,7%)	56,9%; 70,6%	51 (70,8%)	72 (59,5%)	0,124
Hip	58 (30,1%)	23,5%; 36,6%	19 (26,4%)	39 (32,2%)	0,421
Chest	39 (20,2%)	14,5%; 25,9%	23 (31,9%)	16 (13,2%)	**0,003**
Abdomen	30 (15,5%)	10,4%; 20,7%	5 (6,9%)	25 (20,7%)	**0,013**
Leg	3 (1,6%)	0,2%; 3,3%	2 (2,8%)	1 (0,8%)	0,557
Head and Neck	6 (3,1%)	0,6%; 5,6%	0 (0%)	6 (5%)	0,086
Genitalia	54 (28%)	21,6%; 34,4%	20 (27,8%)	34 (28,1%)	≈1,0
Back	16 (8,3%)	4,4%; 12,2%	3 (4,2%)	13 (10,7%)	0,175

The distribution of lesions in areas such as the axilla, groin, hip, chest, and abdomen, highlighting differences between male and female patients, is summarized. The bold values are indicate statistically significant results, with a *p*-value less than 0.05.

### 3.4 Pediatric patients with HS

The pediatric subgroup, consisting of five patients (2.5% of the cohort), had a mean age of 16 ± 0.71 years. The initial diagnosis of HS in this group occurred at an average age of 13.6 ± 1.67 years. The average disease duration was 2.6 ± 1.67 years, and the mean diagnostic delay was 1.6 ± 1.52 years (Table 4).

**Table 4:** Pediatric patient demographics and clinical features.

	All Pediatric Patient (*n* = 5)	Female (*n* = 2)	Male (*n* = 3)
Age	16 ± 0,71 (15–17)	16,5 ± 0,71 (16–17)	15,67 ± 0,58 (15–16)
Age of diagnosis	13,6 ± 1,67 (11–15)	12,5 ± 2,12 (11–14)	14,33 ± 1,15 (13–15)
Duration	2,6 ± 1,67 (1–5)	4 ± 1,41 (3–5)	1,67 ± 1,15 (1–3)
Delay in diagnosis	1,6 ± 1,52 (0–4)	2 ± 2,83 (0–4)	1,33 ± 0,58 (1–2)
BMI	29,23 ± 7,06 (22–38,9)	28,12 ± 8,6 (22–34,2)	29,96 ± 7,8 (24,8-38,9)

Provides detailed demographic information and clinical characteristics of pediatric patients, including age, age at diagnosis, disease duration, and diagnostic delay.

### 3.5 Diagnostic factors for predicting disease severity based on Hurley stages

The Hurley stage distribution was 61.1% in stage I, 24.4% in stage II, and 14.5% in stage III, with males being more likely to be in higher stages (*p* = 0.007). Ordinal logistic regression analysis identified significant predictors of Hurley stage severity in patients with HS. Females were less likely to have higher Hurley stages than males (OR 0.34, 95% CI, 0.164–0.707; *p* = 0.004). A longer disease duration was associated with increased severity (OR 1.04, 95% CI, 0.993–1.090; *p* = 0.093). Anatomical involvement in the inguinal (OR 2.94, *p* = 0.012), hip (OR 2.78, *p* = 0.004), abdomen (OR 2.38, *p* = 0.045), head–neck (OR 6.25, *p* = 0.045), and genital regions (OR 4.76, *p* < 0.001) were significant predictors. The small sample size in some regions, such as the head–neck and legs, may affect reliability and should be interpreted cautiously (Table 5).

**Table 5:** Ordinal logistic regression results for hurley staging.

	Univariate		Multiple		Hierarchical model	
	OR (95% GA)	*p*	OR (95% GA)	*p*	OR (95% GA)	*p*
Body mass index	1,03 (0,974; 1,083)	0,330	0,97 (0,897; 1,038)	0,341		
Diagnosis Age	1,01 (0,986; 1,034)	0,418	1,02 (0,983; 1,055)	0,301		
Delay in Diagnosis	1,04 (0,992; 1,093)	0,100	1 (0,947; 1,062)	0,915		
Disease Duration	1,07 (1,023; 1,118)	**0,003**	1,06 (1,001; 1,126)	**0,048**	1,04 (0,993; 1,09)	0,093
Cigarette	1,02 (1,001; 1,049)	**0,038**	1 (0,966; 1,034)	0,976		
Female Gender (Ref: Male)	0,37 (0,198; 0,703)	**0,002**	0,32 (0,143; 0,732)	**0,007**	0,34 (0,164; 0,707)	**0,004**
Family History: None (Ref: Yes)	1,81 (0,87; 3,765)	0,112	1,83 (0,761; 4,411)	0,177		
Alcohol: None (Ref: Minimum 1) per week	0,77 (0,328; 1,804)	0,546	1,18 (0,41; 3,398)	0,760		
Alcohol: Rarely (Ref: Minimum 1) per week	1,13 (0,446; 2,874)	0,794	0,87 (0,279; 2,722)	0,813		
Comorbidity: None (Ref: Yes)	0,66 (0,377; 1,169)	0,156	1,02 (0,493; 2,108)	0,957		
Axilla Involvement: Yes (Ref: None)	0.74 (0.368; 1.484)	0.394	1.3 (0.531; 3.155)	0.570		
Inguinal Involvement: Yes (Ref: None)	4 (2.028; 7.874)	**<0.001**	2.94 (1.22; 7.194)	**0.016**	2.94 (1.269; 6.803)	**0.012**
Hip Involvement: Yes (Ref: None)	4.17 (2.252; 7.692)	**<0.001**	2.7 (1.333; 5.556)	**0.006**	2.78 (1.377; 5.464)	**0.004**
Chest Involvement: Yes (Ref: None)	1.52 (0.771; 3.003)	0.226	1.43 (0.554; 3.731)	0.455		
Abdomen Involvement: Yes (Ref: None)	2.94 (1.397; 6.098)	**0.004**	2.27 (0.908; 5.78)	0.079	2.38 (1.019; 5.65)	**0.045**
Leg Involvement: Yes (Ref: None)	3.13 (0.378; 25)	0.293	1.64 (0.124; 22.222)	0.704		
Head–Neck Involvement: Yes (Ref: None)	5 (1.065; 22.222)	**0.041**	7.14 (1.073; 47.619)	**0.042**	6.25 (1.043; 38.462)	**0.045**
Genital Involvement: Yes (Ref: None)	5 (2.674; 9.434)	**<0.001**	5 (2.283; 10.87)	**<0.001**	4.76 (2.283; 10)	**<0.001**
Head and Neck Involvement: Yes (Ref: None)	4 (1.541; 10.526)	**0.004**	3.03 (0.862; 10.417)	0.084	2.7 (0.862; 8.696)	0.088

Presents the results of univariate and multiple ordinal logistic regression analyses, identifying significant predictors of disease severity based on the Hurley classification. The bold values are indicate statistically significant results, with a *p*-value less than 0.05.

## 4 Discussion

In this single-center study, we comprehensively analyzed the demographic, clinical, and comorbidity data of patients with HS for approximately 6 years. Globally, the prevalence of HS varies significantly from 0.03 to 4%. Europe and the USA report HS prevalences of approximately 0.8 and 0.2% respectively, whereas the Asia-Pacific region shows a wider variation from 0.01 to 2.2%, indicating a generally lower incidence and prevalence compared to Western countries (6). Research specific to Türkiye indicates a prevalence and clinical presentation of HS comparable to that seen in Western populations, highlighting both demographic and comorbidity patterns distinctive to the region (7, 8). In our study, the male-to-female ratio for HS was approximately 1.7:1. This contrasts with the predominance of females in Europe and North America, where the ratio is approximately 1:3, and the male dominance observed in South Korea, with a ratio of 2:1 (9). These disparities likely arise from genetic, immune, and hormonal differences influenced by geographical and ethnic factors.

The patients had an average age of 34.5 years, were diagnosed at 28.8 years, had a disease duration of 6 years, and had a diagnostic delay of 4.3 years. In contrast, a German cohort reported an average diagnostic delay of 10 years, which exacerbated the disease severity and necessitated more frequent surgical interventions. Such delays, typically not due to patient factors like age or smoking, highlight the need for enhanced clinical awareness (10). Unlike psoriasis, which has a shorter diagnostic delay of approximately 1.6 years (11), HS requires more specific clinical diagnostic criteria. Improved training of healthcare providers on HS diagnostic criteria and initial treatment strategies could reduce these delays, ensuring that evidence-based care is promptly initiated. This is especially crucial for primary care providers, the first contact for many patients with HS. Additionally, a recent web-based survey in Japan suggested that the low reported prevalence of HS might be due to underdiagnosed or misdiagnosed cases, indicating that the actual number of patients could be higher than estimated (12).

Among the patients, 34.4% were obese, with males showing a higher prevalence (36.4%) than females (31%). Smoking was common, with 60.6% of patients being smokers. Male smokers consumed an average of 14 packs per year, significantly more than the 7.2 packs/year reported by females. Research highlights the substantial impact of obesity on HS, particularly through alterations in metabolic biomarkers, whereas smoking has less pronounced effects (13). Approximately 40% of HS patients consume alcohol, and males less likely to abstain compared to females. Effective HS management should include dietary modifications like adopting a Mediterranean diet and reducing high-glycemic foods alongside medical therapy. These changes address symptoms potentially exacerbated by insulin resistance and inflammation (14).

HS often develops in genetically predisposed individuals (15), with 21.8% of our cohort reporting a family history, correlating with earlier disease onset. This supports the findings of Ingram, who noted that about one-third of patients with HS have familial ties, suggesting an autosomal dominant inheritance pattern (16). Although autosomal dominant transmission is typical, monogenic inheritance is rare. Mutations in γ-secretase components such as presenilin 1 and nicastrin disrupt Notch signaling and influence HS phenotypes (17). A genome-wide association study highlighted the involvement of SOX9 and KLF5 in epidermal differentiation and follicular inflammation, offering insights into genetic links with comorbidities and new therapeutic targets for HS (18).

During the active periods, our HS patients frequently experienced pain, discomfort, itching, swelling, and fever, listed in order of decreasing frequency. Flare frequencies varied within our study cohort, with some patients reporting no flares while others experienced multiple episodes monthly or yearly. No significant gender differences were observed in the occurrence of the symptoms. A study found that 83.6% of patients with HS experienced pain during flares, with women and smokers often reporting more severe pain, which intensified with the expansion of affected areas and overall disease severity (19). The variability in HS flare symptoms significantly affects both the physical and emotional well-being of patients, underscoring the need for personalized management strategies (20). Moreover, a disconnect between patient pain experiences and healthcare provider perceptions complicates HS management, emphasizing the need for better communication (21). In a comprehensive study of 1,795 patients with HS, it was shown that HS significantly impacts quality of life, with higher Dermatology Life Quality Index (DLQI) scores in women, which are closely correlated with disease severity and pain levels (22).

Meta-analysis data from countries such as Ireland, the USA, and the UK show that despite higher educational attainment, HS patients face significantly higher unemployment rates compared to their peers (23). Our study further emphasizes this issue, revealing a consistent 5.7% workforce withdrawal due to HS across genders, with a stark contrast in unemployment rates—36.1% in females versus 3.3% in males. These findings underscore the urgent need for improved workplace accommodation, specialized employment, and rehabilitation programs for those affected by HS.

The axilla, affected in 80.8% of patients, is the most commonly involved area in HS, indicative of the disease’s affinity for apocrine gland-rich regions. It was followed by the groin at 63.7% and the hip at 30.1%, with the head and neck least affected at only 3.1%. HS typically affects areas rich in apocrine glands, such as the axillae and inguinal and anogenital regions, but can also be present in regions such as the waist and abdomen. The inflammation characteristic of HS typically surrounds squamous epithelium-lined structures that are likely abnormally dilated hair follicles, indicating a stronger association with hair follicles than apocrine glands (24). Additionally, research shows that HS lesions are not confined to intertriginous zones but can develop in any area containing hair follicles, reinforcing the idea that hair follicles are central to the pathogenesis of HS (25).

Specific data on pediatric HS patients are scarce; it is estimated that less than 2% of HS cases begin before the age of 11 (26). In our study, the pediatric group comprises five patients, with an average age of 16 years and an average diagnostic delay of 1.6 years. HS typically shows a bimodal age distribution, peaking in the late teens and mid-forties. Those with later onset often experience longer diagnostic delays, and nearly half of the pediatric HS patients present with scarring at the time of diagnosis. This underscores the need for increased clinical awareness and early intervention to effectively prevent progression and manage symptoms (27, 28).

HS tarda, observed in the elderly, manifests as late-onset HS beginning after age 60, with four cases in our study, or as persistent HS continuing beyond this age, with three cases in our cohort. Patients with HS tarda often have a significant family history, suggesting a genetic predisposition, and face a higher risk of developing various comorbidities (29).

In our study, 48.2% of patients with HS presented with comorbidities. The most common were psychiatric disorders (19.2%), DM with insulin resistance (14%), and hypertension (9.3%), followed by dyslipidemia (6.7%), cardiovascular diseases (4.7%), and inflammatory bowel disease (IBD) (3.1%). Research underscores a notable link between HS and psychiatric conditions such as schizophrenia, bipolar disorder, depression, anxiety, and substance abuse, underscoring the importance of a multidisciplinary care approach (30). A meta-analysis further revealed significant associations between HS and metabolic issues like hypertriglyceridemia, low HDL cholesterol, and metabolic syndrome, emphasizing the importance of routine cardiovascular screening (31). In a study investigating the relationship between HS and cardiovascular diseases, patients with HS were found to have a 200% higher risk of developing conditions such as heart failure, myocardial infarction, and deep vein thrombosis than healthy individuals, which were further exacerbated by systemic inflammation, obesity, and DM (32). Almuhanna et al. demonstrated that patients with HS have an increased prevalence of rheumatologic conditions such as psoriatic arthritis, ankylosing spondylitis, and rheumatoid arthritis compared to the general population (33). In contrast to our finding of a 4.1% prevalence of SpA, Rondags et al. reported that HS was significantly more prevalent in patients with axial SpA, suggesting that HS might be an extra-articular manifestation of SpA (34). Moreover, a systematic review and meta-analysis showed that patients with HS have increased odds of having CD and UC by 2.12-fold and 1.51-fold, respectively. HS and IBD share clinical, genetic, and immunological characteristics, including cytokine abnormalities like elevated IL-1, IL-6, IL-17, IL-23, and TNF, linking them to a higher prevalence of SpA responsive to TNF inhibitors (35).

In our study cohort, 57.5% of the patients had a history of acne vulgaris. The prevalence of FOT was 6.7% for DCS, 10.9% for AC, and 35.8% for PD. Only five cases met all criteria for FOT, with men showing significantly higher rates of all components. Recent studies indicate that HS patients are substantially more likely to develop PD, with a risk increase of 4.97 to 5.61 times; dissecting cellulitis of the scalp, with a risk 13.38 times higher; and acne vulgaris/conglobata, with a likelihood 1.77 to 5.07 times greater than controls (36). Notably, PD may be associated with an earlier onset and increased severity of HS, suggesting its potential use as an early diagnostic marker in affected individuals (37).

The Hurley classification system, widely recognized for its practicality, was utilized in our study to assess the clinical severity and long-term progression of HS, aligning our findings with global standards (38). We observed that many patients with early HS were initially misdiagnosed with conditions such as sebaceous or epidermoid cysts, folliculitis, and furunculosis because of similarities in early symptoms. This highlights a significant risk of misdiagnosis in patients with mild HS, who often do not exhibit distinctive lesions like sinus tracts found in more advanced stages. Furthermore, studies have found a significant positive correlation between higher Hurley stages and increased severity scores, such as DLQI and IHS4, underscoring the importance of accurate early diagnosis and the effectiveness of the Hurley system in reflecting the clinical reality of HS progression (39, 40).

In our study, ordinal regression analysis revealed that HS disease severity was significantly correlated with disease duration, cigarette consumption, male gender, and atypical localization areas such as the legs and posterior neck. HS severity tends to be greater in men, potentially due to elevated androgen levels which influence sebaceous gland activity, alongside a higher prevalence of smoking and delayed healthcare-seeking behavior, all of which collectively exacerbate disease outcomes. Furthermore, atypical localization of HS, like on the scalp or posterior neck, is linked with increased disease severity, primarily due to diagnostic delays and the reduced efficacy of treatments in these less commonly affected areas. These results align with previous research, which highlights factors such as increased age, male gender, and obesity in worsening HS severity (41). Importantly, we differentiated risk factors into modifiable factors, such as smoking, obesity, diet, and stress management, and non-modifiable factors, including genetic predisposition, age of onset, and hormonal influences related to gender. This distinction is crucial for developing personalized management plans that significantly enhance the quality of life for patients with this chronic and debilitating condition.

## 5 Conclusion

Disease severity in HS was significantly associated with disease duration, the number of cigarette packs per year, male gender, and atypical localization. Geographic and cultural factors can influence the prevalence, severity, and management of HS by affecting lifestyle choices, healthcare access, dietary habits, and social attitudes towards skin conditions. This necessitates tailored approaches for effective treatment and support. The relationship between HS and its comorbidities is bidirectional, while late diagnosis can lead to irreversible skin damage, and comorbidities can also exacerbate HS. Therefore, effective management of HS requires multidisciplinary routine screening measures for the early detection and prevention of comorbidities, including psychiatric disorders, cardiovascular conditions, and metabolic syndrome, as its complications extend across multiple organ systems.

## 6 Limitations

Our study had several limitations, including potential recall bias due to its retrospective design. As a single-center study, the findings might not be broadly applicable to other populations. The small sample size for atypical lesion locations could have restricted our ability to detect significant associations. Furthermore, while the Hurley classification system is a well-recognized method for assessing HS clinical severity, its static nature might not fully reflect the dynamic progression of the disease or the response to treatment, which could lead to an incomplete evaluation of disease severity over time. Additionally, the loss of some patients to follow-up limited our capacity to assess the relationship between treatment options and disease severity.

## Data Availability

The raw data supporting the conclusions of this article will be made available by the authors, without undue reservation.
